# Beyond Routine Monitoring: A Comprehensive Review of Direct Oral Anticoagulants and the Role of Coagulation Profiles in Their Management

**DOI:** 10.7759/cureus.80469

**Published:** 2025-03-12

**Authors:** Jayalekshmi Jayakumar, Fiqe Khan, Meher Ayyazuddin, Davin Turku, Manasa Ginjupalli, Aju Kalaivani Babu, Srinishant Rajarajan, Mrunanjali Gaddam, Vikash Kumar, Asmat Ullah

**Affiliations:** 1 Internal Medicine, The Brooklyn Hospital Center, New York, USA; 2 Internal Medicine, Combined Military Hospital (CMH) Lahore Medical College and Institute of Dentistry, Lahore, PAK; 3 Internal Medicine, Allegheny General Hospital, Pittsburgh, USA

**Keywords:** apixaban, doac, doac and ptt, monitoring doac, oral anticoagulants

## Abstract

Direct oral anticoagulants (DOACs) have revolutionized anticoagulation therapy, providing effective and safe management of thrombosis and related conditions. As their use continues to grow, accurately monitoring their effects is essential to achieve optimal patient outcomes. Traditional coagulation tests, such as prothrombin time (PT) and activated partial thromboplastin time, have long been used to evaluate clotting function and bleeding risk in patients on anticoagulants. However, these standard tests often fall short with DOACs due to complex interactions between the drugs and the assays. While PT offers some insight into coagulation, its reliability for drugs like apixaban, one of the most commonly prescribed DOACs, remains debated. This limitation underscores the need for alternative monitoring strategies, such as the modified diluted PT, which shows promise in providing more accurate assessments of DOAC levels. This review discusses the pharmacokinetics of DOACs, their impact on standard coagulation tests, and various factors - such as liver disease and drug interactions - that complicate these assessments. Additionally, it highlights the importance of incorporating specific assays, including anti-factor Xa activity and dilute thrombin time, for precise anticoagulation management. By synthesizing current evidence, this review aims to identify improved methods for monitoring DOAC therapy, guide clinicians in optimizing anticoagulation treatment, and ultimately enhance patient outcomes.

## Introduction and background

Blood clotting studies are crucial for assessing an individual’s coagulation status and determining the risk of bleeding or thrombosis. Two fundamental tests in this evaluation are prothrombin time (PT) and partial thromboplastin time (PTT). PT measures the time required for blood to clot, primarily assessing the extrinsic pathway of the coagulation cascade, with normal values ranging from nine to 13 seconds. In contrast, PTT evaluates the intrinsic and common coagulation pathways, focusing on factors VIII, IX, XI, XII, and fibrinogen, with normal values between 25 and 35 seconds [1]. The INR adjusts for variations in PT measurements caused by differences in devices and assays [2].

Prolonged PT may indicate deficiencies in clotting factors such as fibrinogen, factors V, VII, X, and prothrombin and may suggest conditions like liver disease, vitamin K deficiency, or the presence of anticoagulants [1]. Additionally, a recent study identified a positive correlation between PT and the decreased frequency of CD39+CD73+ B cell subsets in patients with systemic lupus erythematosus [3]. While prolonged PTT can indicate deficiencies or the presence of inhibitors, a shortened PTT is often associated with an elevated risk of thrombosis due to increased factor VIII levels. Activated PTT (aPTT), which measures the same factors as PTT but includes an activator, provides a more specific diagnostic range [4,5].

Anticoagulants are drugs designed to inhibit the coagulation pathway [6]. Heparin, the first anticoagulant, was discovered in the 1910s, followed by warfarin, with low molecular weight heparin (LMWH) and fondaparinux being developed toward the end of the 20th century [7]. Direct oral anticoagulants (DOACs), a more recent class of anticoagulants, offer an improved safety profile and have become a preferred option for preventing and managing thrombosis [8]. While PT offers some insight into the effects of DOACs, its reliability, especially for apixaban, is often debated, rendering it inadequate for monitoring these medications [9].

This underscores the need for more reliable methods to monitor coagulation in patients using DOACs. A promising alternative is the modified diluted PT (mdPT), a new reagent for PT that shows potential for improving the accuracy of DOAC monitoring [10].

This article provides a comprehensive review of current coagulation tests, their applicability to DOAC monitoring, and emerging diagnostic methods that could enhance precision and effectiveness in clinical anticoagulation management.

## Review

Mechanism of action of DOACs

DOACs are mainly classified into two categories: oral direct factor Xa inhibitors (rivaroxaban, apixaban, and edoxaban) and direct thrombin inhibitors (dabigatran) [11]. These small-molecule anticoagulants work by binding to the catalytic site of either factor Xa or thrombin, blocking their ability to cleave and activate substrates. This targeted mechanism offers a broader therapeutic window, allows for more flexible monitoring, and minimizes the risk of drug-drug interactions [12].

**Figure 1:**
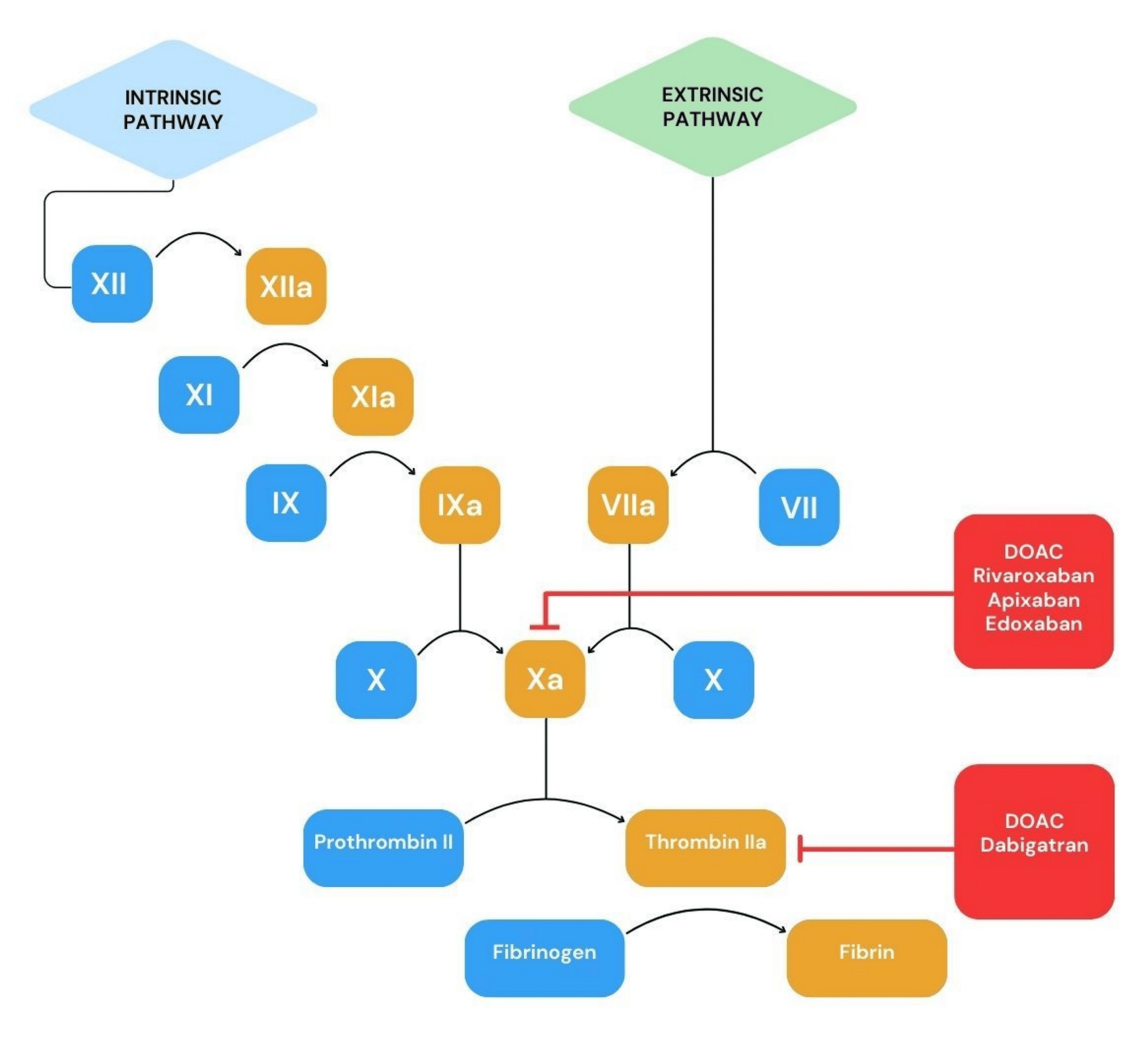
Mechanism of action of DOACs DOACs, direct oral anticoagulants Created by Meher Ayyazuddin using Canva (Canva Pty. Ltd., Sydney, NSW, Australia)

The development of dabigatran involved incorporating ethyl-ester and hexyl-oxy-carbonyl carbamide hydrophobic side chains into α-N-(2-naphthylsulfonylglycyl)-4-amidino-DL-phenylalaninepiperidide, resulting in an orally absorbed prodrug now widely used in clinical practice [13]. Unlike other DOACs, dabigatran directly inhibits thrombin, while factor Xa, a serine protease positioned upstream in the coagulation cascade, presents another key target for anticoagulation therapy (Figure 1) [14,15].

Dabigatran functions as a selective, competitive inhibitor of thrombin (factor IIa), whereas rivaroxaban, apixaban, and edoxaban selectively and competitively inhibit activated factor Xa. Apixaban and rivaroxaban are known for their relatively short half-life and rapid onset of anticoagulant activity, enabling them to quickly reach peak plasma concentrations without the need for overlapping therapy with parenteral anticoagulants. However, edoxaban and dabigatran require initial overlap with parenteral anticoagulants. Once discontinued, DOACs are rapidly eliminated from the body, although renal function can influence this clearance process (Table 1) [16]. 

**Table 1:** Summary of the mechanism of action, pharmacokinetics, and uses of different DOACs AF, atrial fibrillation; BID, bi-daily; CrCl, creatinine clearance; DOACs, direct oral anticoagulants; LMWH, low molecular weight heparin; NVAF, non-valvular atrial fibrillation; VTE, venous thromboembolism; Xa, factor Xa Table created by Davin Turku

DOAC	Mechanism of action	Specific indication	Half-life	Duration of action	Dose adjustment in renal failure	Metabolism	Antidote	Need for bridging therapy
Apixaban	Selective factor Xa inhibitor	Non-valvular AF (stroke prevention)	12 hours	~12 hours	Reduce to 2.5 mg BID if serum creatinine ≥1.5 mg/dL with age ≥80 years or weight ≤60 kg (in NVAF); avoid if CrCl <15 mL/min	Mainly metabolized by CYP3A4 and CYP3A5; some renal elimination	Andexanet alfa	No
		VTE treatment and prevention						
		Post-op VTE prophylaxis						
Rivaroxaban	Selective factor Xa inhibitor	Non-valvular AF (stroke prevention)	5-9 hours	~24 hours	Avoid if CrCl <15 mL/min for NVAF; reduce to 15 mg daily if CrCl 15–50 mL/min for NVAF; avoid if CrCl <30 mL/min for VTE	Primarily metabolized by CYP3A4 and CYP3A5; some renal elimination	Andexanet alfa	No
		VTE treatment and prevention						
		Post-op VTE prophylaxis						
Edoxaban	Selective factor Xa inhibitor	Non-valvular AF (stroke prevention)	10-14 hours	~24 hours	Reduce to 30 mg daily if CrCl 15-50 mL/min; avoid if CrCl <15 mL/min or >95 mL/min (NVAF)	Mostly excreted unchanged by kidneys; minimal metabolism by CYP3A4	None	Yes (heparin or LMWH)
		VTE treatment after initial heparin therapy						
Dabigatran	Direct thrombin (factor IIa) inhibitor	Non-valvular AF (stroke prevention)	12-17 hours	~22 hours	Reduce to 75 mg BID if CrCl 15-30 mL/min (NVAF); avoid if CrCl <15 mL/min; not recommended if CrCl <30 mL/min for VTE	Metabolized by CYP3A4; conjugated by UGT1A1; primarily excreted unchanged by kidneys	Idarucizumab	Yes (heparin or LMWH)
		VTE treatment and prevention after initial heparin therapy						

Compared to vitamin K antagonists (VKAs) and heparin, DOACs offer several advantages, including reduced monitoring requirements and less frequent follow-ups. They also provide a more immediate onset and offset of anticoagulant effects, making them particularly useful for periprocedural management and acute bleeding control. Furthermore, DOACs have fewer interactions with drugs and dietary factors, enhancing their safety profile and convenience [17].

Uses of DOACs in various diseases

**Figure 2:**
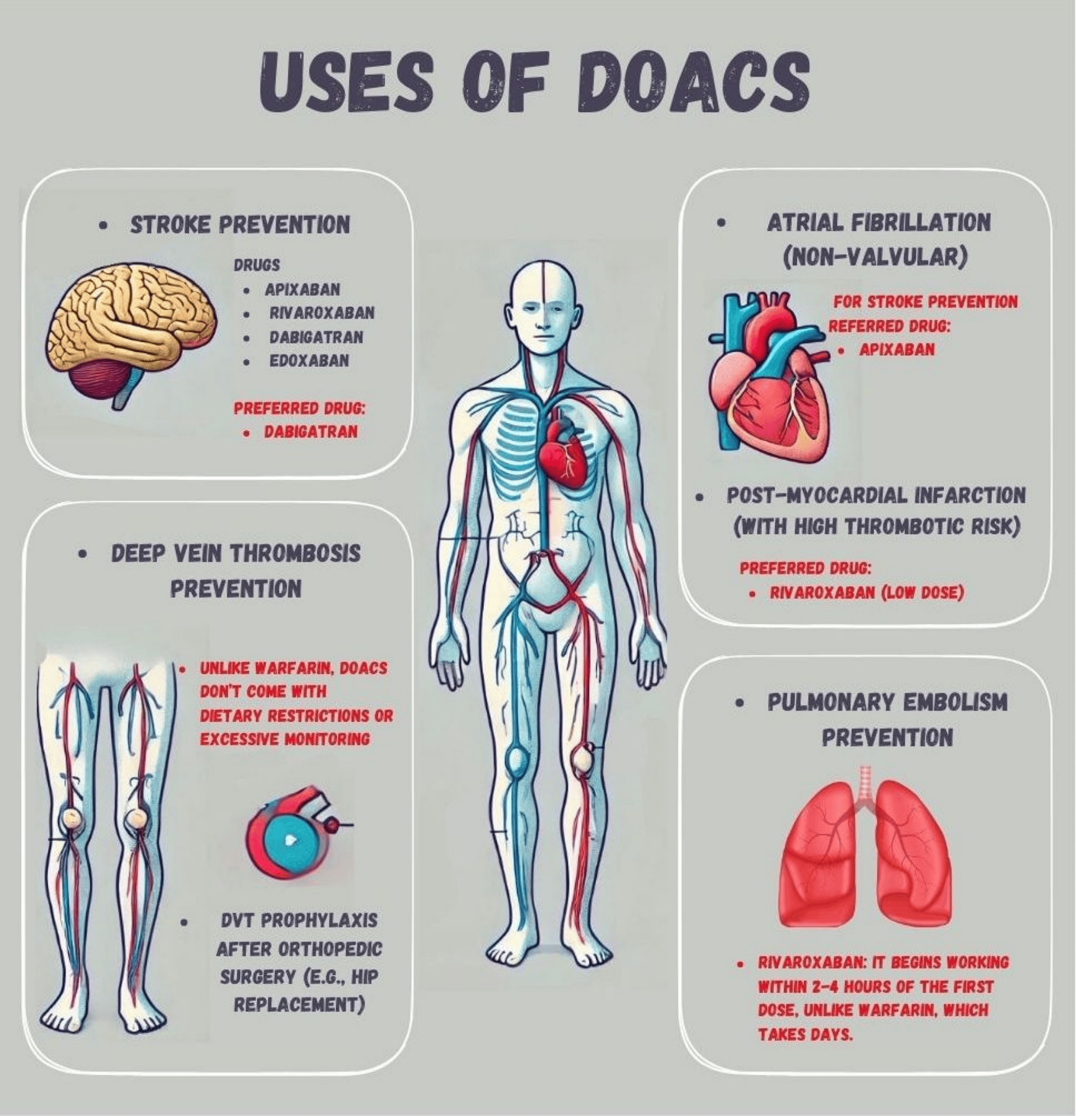
Uses of DOACs DOACs, direct oral anticoagulants; DVT, deep vein thrombosis Created by Meher Ayyazuddin using Canva (Canva Pty. Ltd., Sydney, NSW, Australia)

DOACs have gained significant popularity in recent years due to their favorable safety profile and effectiveness. Their broad therapeutic range, predictable pharmacokinetics and pharmacodynamics, rapid onset of action, availability of antidotes, minimal side effects, and limited interactions with other drugs or foods make them suitable for use in various diseases (Figure 2) [16,17].

Stroke Prevention in Atrial Fibrillation (AF)

The management and outcomes of acute ischemic stroke have significantly improved, partly due to the increasing use of DOACs in patients with AF [18]. DOACs, including dabigatran, rivaroxaban, apixaban, and edoxaban, are approved by the FDA for stroke prevention in non-valvular AF. They are also effective in reducing the risk of systemic embolism, deep vein thrombosis (DVT), and pulmonary embolism (PE) in non-valvular AF [19]. Phase III clinical trials have demonstrated that these agents are at least as effective and safe as warfarin. Notably, DOACs offer a substantial advantage by reducing the risk of intracranial hemorrhage by up to 50% compared to warfarin [20]. However, this benefit comes with an increased risk of GI bleeding, particularly with dabigatran 150 mg twice daily, rivaroxaban, and higher-dose edoxaban [21].

A nationwide French cohort study confirmed that DOACs provide superior safety and effectiveness compared to VKAs in patients with non-valvular AF at high risk for GI bleeding [22]. Among the DOACs, apixaban was associated with the lowest risk of major bleeding and GI bleeding. In terms of stroke and systemic embolism prevention, apixaban demonstrated a reduced risk compared to rivaroxaban and a similar risk profile to dabigatran [22].

For patients classified as high-risk according to the CHA₂DS₂-VASc score, prior use of DOACs significantly increased the likelihood of successful and complete reperfusion following a stroke. Conversely, for patients with intermediate to low-risk scores, neither prior DOAC nor warfarin use influenced outcomes. Regardless of the total CHA₂DS₂-VASc score, individuals with specific risk factors such as congestive heart failure or left ventricular (LV) dysfunction, hypertension, age >75 years, or female sex experienced similar benefits from prior DOAC use [23].

Venous Thromboembolism (VTE)

Anticoagulation plays a critical role in managing VTE and ensuring its long-term prevention. Dabigatran has demonstrated non-inferiority in preventing VTE recurrence, with statistically significant results and a lower risk of bleeding events compared to traditional therapies [24]. Studies examining various doses of apixaban, edoxaban, and rivaroxaban against standard LMWH therapy have shown these agents to be effective in preventing recurrent symptomatic VTE while also reducing the risk of major bleeding events [24,25].

Additionally, an extended placebo-controlled randomized controlled trial highlighted rivaroxaban’s efficacy. Patients receiving rivaroxaban 20 mg once daily for six or 12 months experienced a significantly lower risk of recurrent VTE compared to placebo (hazard ratio = 0.18; 95% CI: 0.09-0.39; p < 0.001), with a comparable risk of bleeding [24,26]. The efficacy of dabigatran in comparison with placebo is similar to that of rivaroxaban versus placebo and warfarin versus placebo or control [27-29].

Although major bleeding rates were similar across all groups, dabigatran notably reduced major or clinically relevant non-major bleeding (p< 0.001). The RE-SONATE trial further validated dabigatran’s effectiveness in lowering recurrent VTE events (p < 0.001), albeit with a slightly increased risk of bleeding compared to placebo [25].

DVT

Research continues to explore the most effective first-line anticoagulant for DVT [30]. A retrospective cohort study comparing DOACs with warfarin found that patients treated with DOACs experienced significantly lower rates of progression to PE compared to those on warfarin. However, no significant difference was noted in the progression to proximal DVT between the two groups [30].

The reason behind this disparity - where DOACs appear more effective in preventing PE but not proximal DVT progression - remains unclear. This observation underscores the need for further investigation into the mechanisms of anticoagulation concerning distal DVT propagation versus embolization. Despite this uncertainty, patients treated with DOACs demonstrated lower rates of progression to PE and a reduced incidence of major bleeding, suggesting that DOACs may be more effective than warfarin in treating distal DVT [30].

The adoption of DOACs as the preferred first-line treatment for DVT has also facilitated outpatient care, now considered the standard approach for most patients with uncomplicated DVT [31,32]. Determining the appropriate duration of anticoagulation therapy for lower extremity DVT involves balancing the risk of bleeding against the likelihood of recurrence with or without continued treatment. Expert consensus recommends extended anticoagulant therapy when the risk of VTE recurrence exceeds 5% annually or 15% over five years, as the benefits outweigh the risks [33]. Given the lower bleeding risk associated with DOACs, these thresholds may be adjusted to less than 3% per year, particularly when prophylactic doses of rivaroxaban or apixaban are employed [34].

PE

The treatment landscape for PE has broadened to include DOACs. While LMWH bridging to warfarin has traditionally been the most common treatment for older adult PE patients, DOACs are increasingly being used [35]. Although extensively studied for AF, their use in PE, particularly among older patients, is less well documented. However, a cohort study demonstrated that the safety and efficacy of DOACs in treating PE are consistent across age groups, providing reassurance about their use in older populations [36].

VTEs, including DVT and PE, frequently occur following total knee arthroplasty and total hip arthroplasty [37,38]. Recent studies suggest that aspirin is an effective prophylactic agent for VTE after major orthopedic surgery. A meta-analysis comparing standardized aspirin use with DOACs, such as rivaroxaban, found no significant difference in efficacy or safety. This finding indicates that aspirin is as effective as rivaroxaban in preventing VTEs, including PE and DVT, with similar safety profiles [39].

Rivaroxaban has also been shown to be non-inferior to enoxaparin/warfarin (VKA) regimens in preventing recurrent symptomatic VTE, with comparable bleeding rates (p < 0.001) in the EINSTEIN-PE trial. Additional studies have validated rivaroxaban’s efficacy and safety by directly comparing it with LMWH/VKA in patients with acute PE, confirming its viability as an alternative for VTE management [40].

LV Thrombus

The efficacy and safety of DOACs in managing LV thrombus following acute myocardial infarction remain under investigation. Traditionally, VKAs have been the standard treatment due to the significant risk of systemic embolism associated with LV thrombus [41]. However, emerging evidence suggests that DOACs may offer a superior alternative. A meta-analysis comparing DOACs with warfarin indicated that DOACs were associated with higher rates of LV thrombus resolution, suggesting their potential as a reasonable alternative to warfarin in this setting [41].

Rivaroxaban has demonstrated faster LV thrombus resolution and a reduced likelihood of ischemic stroke compared to VKAs, while apixaban and dabigatran have shown at least equivalent efficacy [42]. Despite these encouraging findings, the limited availability of robust clinical trial data highlights the need for further research to fully understand the effects of DOACs in this context.

Another meta-analysis found that DOACs nearly doubled the likelihood of thrombus resolution compared to VKAs (pooled OR: 1.95, 95% CI: 1.25-3.04, p = 0.003, I² = 0%) and reduced the risk of systemic embolism by 70% (pooled OR: 0.30, 95% CI: 0.12-0.75, p = 0.01, I² = 0%) [43]. Additionally, DOACs were associated with a 54% lower risk of bleeding (pooled OR: 0.46, 95% CI: 0.26-0.84, p = 0.01, I² = 0%) and a 63% lower risk of reaching the composite safety and efficacy outcome compared to VKAs [43]. These findings underscore the favorable safety and efficacy profile of DOACs in managing LV thrombus, highlighting their potential as a preferred treatment option.

Factors affecting PT and aPTT levels in DOAC usage

Several factors can influence PT and aPTT levels in patients receiving DOAC therapy. These factors include:

*Drug* 

The impact of DOACs on coagulation tests like PT and aPTT varies depending on the specific medication. Dabigatran, a direct thrombin inhibitor, can prolong aPTT, but a normal aPTT result does not exclude its presence. Conversely, direct factor Xa inhibitors, such as rivaroxaban, edoxaban, and apixaban, generally do not prolong aPTT. While rivaroxaban and edoxaban can prolong PT, apixaban has minimal effects on PT values. Dabigatran, however, does not significantly affect PT levels [44-46].

Routine coagulation tests, such as aPTT and PT/INR, do not reliably correlate with plasma DOAC concentrations. Even patients receiving full anticoagulation with DOACs, particularly apixaban, may show normal or near-normal test results. This highlights the need for specialized assays, such as anti-factor Xa levels or dilute thrombin time, to accurately assess DOAC activity [47].

Drug Concentration 

DOACs generally act as nonspecific inhibitors during mixing tests for aPTT and PT, with stronger inhibition seen at higher concentrations [44]. The correlation between dabigatran and aPTT prolongation is poor; normal aPTT results may be obtained even at low concentrations, making aPTT an unreliable indicator for dabigatran. PT is similarly insensitive to dabigatran.

In contrast, rivaroxaban and edoxaban show concentration-dependent prolongation of both aPTT and PT, although the sensitivity of these tests is insufficient for accurately reflecting plasma concentrations. Therefore, more specific assays, such as anti-factor Xa tests, are necessary for precise measurement of DOAC activity [47].

Reagent 

The sensitivity of coagulation assays to DOACs varies significantly among different reagents and drugs. PT and aPTT reagents are generally more responsive to edoxaban and rivaroxaban than to apixaban, with no reagent reliably detecting apixaban [45].

For rivaroxaban and edoxaban, aPTT reagents show some dose-dependent responses, but PT reagents tend to be more sensitive. However, studies often use drug-enriched normal plasma, which may not accurately represent patient samples. Dabigatran’s effect on aPTT also varies across reagents, complicating interpretation [45,47].

Patients with identical DOAC plasma concentrations may exhibit different PT or aPTT results, making it challenging to interpret coagulation tests accurately. Normal PT or aPTT results do not necessarily exclude high plasma concentrations of DOACs, especially when poorly responsive reagents are used. Therefore, relying on PT or aPTT alone to assess DOAC levels can be misleading, necessitating the use of more specific assays such as anti-Xa activity or dilute thrombin time [48].

Liver Disease

The liver is responsible for producing many clotting factors, making PT a common tool for assessing liver function. Liver diseases such as cirrhosis, hepatitis, or liver failure can impair the synthesis of these factors, resulting in prolonged PT. Similarly, aPTT and other coagulation tests may indicate liver conditions that affect coagulation factor production.

When interpreting abnormal coagulation results, the full clinical context, including the patient’s medical history and any contributing factors, must be considered. For patients with liver disease, abnormal PT and aPTT values may indicate compromised liver function. These findings should be integrated with other diagnostic information to provide an accurate assessment [49].

Interference With Other Drugs

The metabolism of individual DOACs varies but typically involves the cytochrome P450 isoenzyme system (CYP) and/or permeability glycoprotein (P-gp) [50]. Drug interactions often occur when substances affect CYP450 enzymes or P-gp activity, but some interactions may also impair hemostasis [50].

The concomitant use of DOACs with other anticoagulants, such as heparin, warfarin, or other DOACs, can increase the risk of bleeding. Antiplatelet agents like aspirin, clopidogrel, and ticagrelor also elevate bleeding risk when combined with DOACs. For instance, ticagrelor can increase dabigatran levels, enhancing its anticoagulant effect.

Additionally, drugs such as amiodarone, which moderately inhibits P-gp, can raise DOAC concentrations. Macrolides like clarithromycin and erythromycin, which inhibit both CYP3A4 and P-gp, may also elevate DOAC levels. In contrast, rifampicin decreases DOAC blood levels and reduces their efficacy.

Close monitoring is essential when combining these drugs to detect signs of thrombosis or bleeding. Furthermore, antifungals, anti-seizure medications, and antidepressants should be evaluated for potential interactions before being used alongside DOACs [51].

Methods of assessing bleeding risk

Evaluating a patient’s risk of bleeding is essential when deciding whether to initiate antithrombotic therapy and ensuring their safety during treatment [52]. Bleeding risk assessment can be performed through clinical judgment, considering the patient’s history, physical examination, and comorbidities. However, this approach is highly variable between providers and lacks quantifiability. To address this, standardized scoring systems have been developed to provide a more consistent assessment based on defined criteria.

Two major risk stratification tools are the HAS-BLED and ORBIT scores. The HAS-BLED score, introduced in 2010, was designed to assess bleeding risk in patients receiving anticoagulants for AF. It is an acronym that stands for Hypertension, Abnormal renal/liver function, Stroke, Bleeding history or predisposition, Labile INR, Elderly (age >65 years), and Drugs/alcohol concomitantly, with a maximum score of nine points [53]. The ORBIT score, developed in 2015, aimed to provide a more comprehensive bleeding risk assessment. It includes five components: older age, reduced hemoglobin/hematocrit or anemia, bleeding history, impaired kidney function, and treatment with antiplatelet agents, with a total score ranging from zero to seven points [54].

Although the HAS-BLED score remains the most widely used bleeding risk assessment tool, no universally accepted “gold standard” model exists [53]. Concerning bleeding risk across different DOACs, data do not indicate one agent as significantly more hazardous than others. However, recent studies suggest that rivaroxaban is associated with a higher bleeding risk compared to apixaban, while dabigatran has not demonstrated a significant increase in bleeding risk relative to other DOACs [55].

Advanced assays for monitoring DOACs

The limitations of standard coagulation assays have prompted the development of advanced diagnostic methods to accurately assess DOAC activity. Some of the most promising assays include chromogenic anti-factor Xa assays, liquid chromatography-mass spectrometry (LC-MS/MS), modified dilute thrombin time (mdTT), and ecarin clotting time (ECT).

The chromogenic anti-factor Xa assay, which employs a LMWH-calibrated agent, is particularly useful for ruling out clinically relevant concentrations of rivaroxaban and apixaban in emergency situations. However, it is not suitable as a universal method for quantitative measurement of these drugs [56]. LC-MS/MS is regarded as the gold standard for accurately measuring plasma concentrations of all DOACs. This technique directly quantifies drug molecules in pretreated plasma without requiring DOAC-specific calibration, making it a precise and reliable assessment tool [57].

In patients with non-valvular AF receiving apixaban, edoxaban, or rivaroxaban, certain coagulation parameters show age-related differences. However, the mdTT offers a consistent response to drug concentration across all age groups, making it a dependable parameter for monitoring these anticoagulants [58]. ECT is a specialized assay designed to measure the anticoagulant activity of direct thrombin inhibitors like dabigatran by evaluating their pharmacodynamic and pharmacokinetic properties. While ECT offers high specificity and the ability to directly reflect thrombin inhibition, its disadvantages include limited availability, lack of standardization, and reduced applicability for other anticoagulants [59].

## Conclusions

While DOACs have revolutionized thrombosis management, their monitoring remains a complex challenge. Traditional coagulation tests like PT and aPTT are inadequate for accurately assessing DOAC activity, necessitating the use of more specialized assays such as mdPT, anti-Xa assays, and dilute thrombin time. As the clinical use of DOACs continues to expand, adopting these advanced monitoring strategies is essential to ensure therapeutic efficacy and patient safety.

Furthermore, understanding the influence of factors such as liver disease, drug interactions, and drug concentration on coagulation profiles is vital for refining DOAC management. By integrating precise diagnostic tools with a comprehensive understanding of pharmacokinetics and patient-specific factors, healthcare providers can optimize DOAC therapy, minimizing risks and improving patient outcomes. This ongoing evolution in anticoagulation management underscores the importance of continuous research and innovation to enhance patient care. Future directions, including genetic testing for DOAC efficacy and large-scale clinical studies, will be pivotal in guiding the effective application of these novel agents.
